# Inflammatory myofibroblastic tumor of the lung- a case report

**DOI:** 10.1186/1749-8090-5-55

**Published:** 2010-07-20

**Authors:** Chien-Kuang Chen, Chia-Ing Jan, Jian-Shun Tsai, Hsu-Chih Huang, Pin-Ru Chen, Yu-Sen Lin, Chih-Yi Chen, Hsin-Yuan Fang

**Affiliations:** 1 Division of Thoracic Surgery, Department of Surgery, China Medical University Hospital, China Medical University, Taichung, Taiwan; 2 Department of Pathology, China Medical University Hospital, China Medical University, Taichung, Taiwan

## Abstract

A 45-year-old man presented with a six-month history of progressive dyspnea with productive cough and wheezing. The patient was a heavy smoker and had a history of tongue cancer, hypertension, and asthma. Chest X-ray and computed tomography showed a mass lesion in the left hilar region and total collapse of the upper left lobe of the lung. Bronchoscopy revealed a whitish solid tumor obstructing the left upper lobe bronchus. Positron emission tomography showed increased tracer uptake in the lesion. A thoracoscopic lobectomy of the left upper lobe of the lung was performed. The final pathologic diagnosis was inflammatory myofibroblastic tumor.

## Introduction

Inflammatory myofibroblastic tumor (IMT) of the lung, also known as plasma cell granuloma or inflammatory pseudotumor, is a rare disease entity [1]. Diagnosis of IMT is difficult to establish before surgery because of its diversified radiologic manifestations. This tumor can be cystic or homogeneous, endobronchial or parenchymal with or without clear margins [2]. Complete surgical resection is the treatment of choice not only to exclude malignancy but also to achieve a good prognosis [3,4]. We report a case of inflammatory myofibroblastic tumor that was successfully removed by thoracoscopic lobectomy.

## Case report

**Figure 1:**
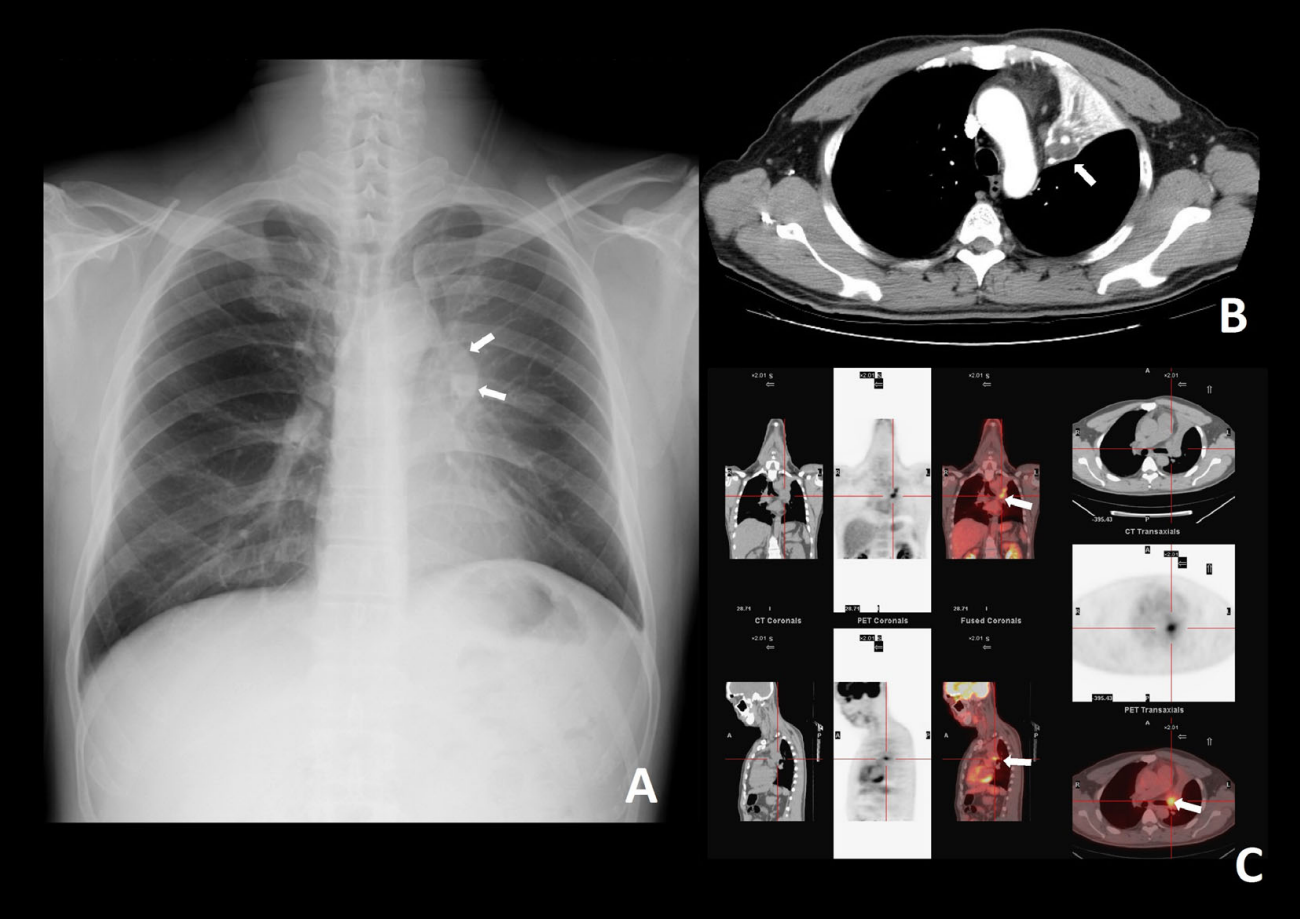
**(A) Chest plain film**. A protruding mass shadow is seen in the left hilar region. The shadow of the left bronchus stops at the mass. Costodiaphragmatic angles are clear. There is increased density over the left lung field with elevation of the left side of the diaphragm. These findings are indicative of a hilar mass obstructing the bronchus with collapse of the left upper lobe of lung. (B) Contrast computed tomography (CT) image, distal part of the tumor. The distal bronchus is dilated and filled with secretions. The margin between the lung parenchyma and tumor is indistinct. (C) Positron emission tomography (PET) and CT, proximal part of the tumor. An endobronchial tumor with high tracer uptake and clear margins is visible.

**Figure 2:**
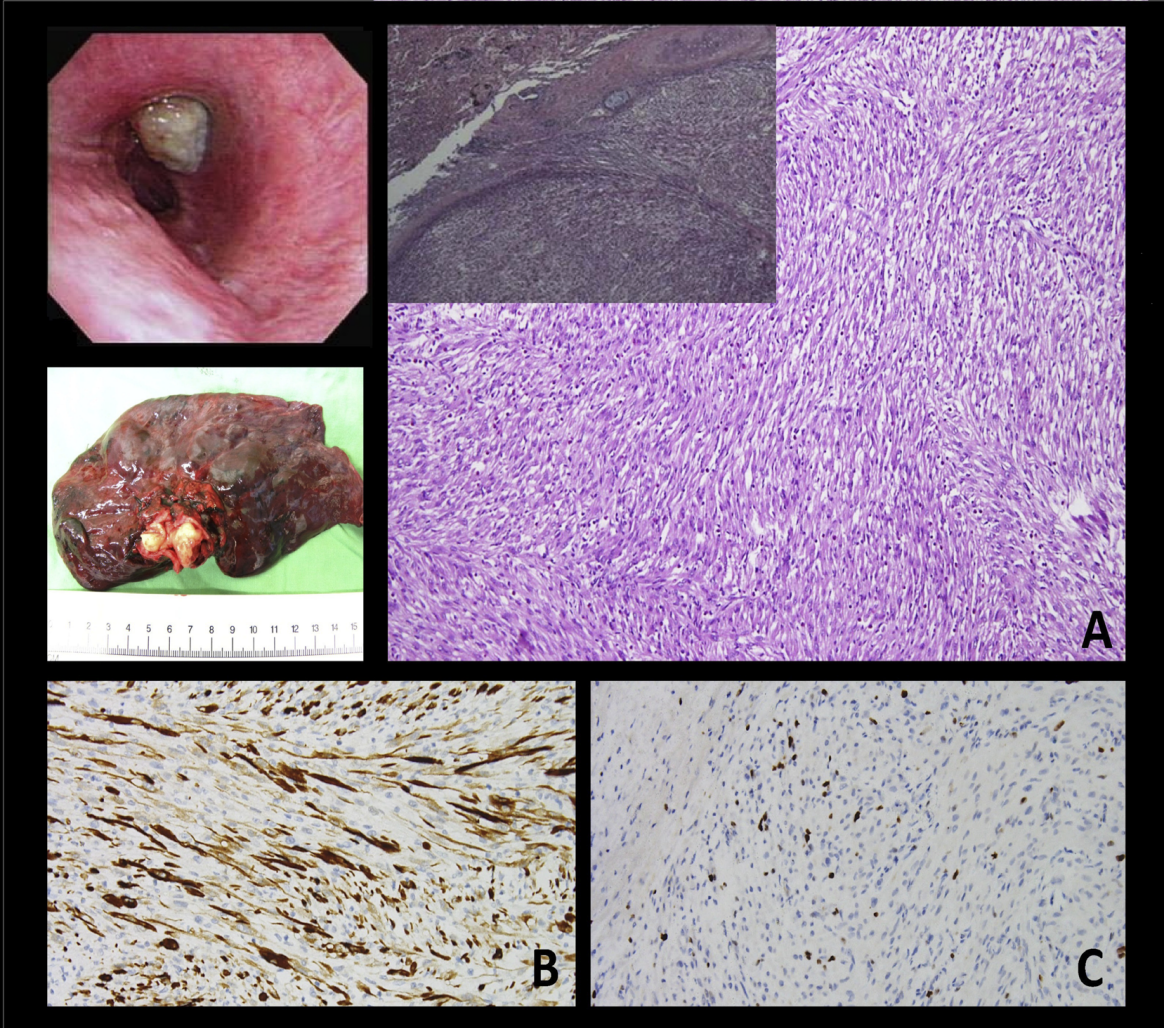
**Bronchoscopic exam shows a whitish tumor obstructing the left upper bronchus**. Gross. The tumor impacted the whole bronchus with clear margins. Microscopically, the biopsy specimen is composed of spindle cells with fibroblastic and myofibroblastic differentiation arrayed in fascicles. (A) The tumor is mostly limited within the bronchi. In a few foci, pushing of tumor margin to the lung parenchyma is noted (×20; ×100). Immunohistochemical study demonstrated (B) vimentin (+) (×200), and (C) cytokeratin (focal +), (×200).

A 45-year-old man presented with a 6-month history of progressive dyspnea with productive cough and wheezing. The patient had a history of smoking (1 pack per day for 20 years), hypertension and asthma, which was under regular medical control. He also had a history of tongue cancer (squamous cell carcinoma, pT2N0M0, stage II) for which he underwent wide excision of the right side of the tongue and modified neck lymph node dissection five years prior to this presentation. Chest plain film showed a protruding mass shadow in the left hilar region. Costodiaphragmatic angles were clear. There was increased density over left lung field with elevation of the left side of the diaphragm. These features were indicative of a hilar mass obstructing the bronchus with collapse of the upper left lobe of the lung (Fig. 1A). Contrast enhanced computed tomography (CT) showed a hilar mass measuring approximately 35 mm × 28 mm × 15 mm and a collapsed left upper lobe of the lung. There was weak enhancement in the arterial phase. The endobronchial part of the tumor had clear margins along the bronchus of the upper left lobe of the lung. The distal part of the tumor had indistinct margins along the lung parenchyma. The distal bronchus was dilated and filled with secretions. There was no mediastinal lymphadenopathy (Fig. 1B). Bronchoscopy revealed a whitish tumor obstructing the left upper bronchus (Fig. 2). Biopsy specimens of the tumor taken during the bronchoscopic examination showed evidence of smooth muscle cell proliferation with focal abnormal mitosis. A smooth muscle cell tumor of malignant potential was considered. Positron emission tomography (PET) showed increased fluorodeoxyglucose (FDG) uptake in the lesion (Fig. 1C).

The tumor involved the upper left lobe of the lung and obstructed the bronchus. The patient underwent a thoracoscopic lobectomy under general anesthesia with double lumen endotracheal tube placement. The vessels of the left upper lobe were divided and ligated using an endoscopic autostapling device. The bronchus of the upper left lobe was opened by endoscissor. The cutting margin was checked by examination of frozen sections to ensure that the resection was clear. The upper left lobe of lung was removed through a port with extended skin incision 5 cm at the anterior 5th intercostal space. The orifice of the bronchus was sutured with standard instrumentation through the utility incision.

The resected tumor was white and elastic, measuring 3.5 cm × 2.5 cm × 1.5 cm in size. It impacted the whole bronchus of the left upper lobe (Fig. 2). Microscopic examination revealed a mixture of spindle cells showing fibroblastic and myofibroblastic differentiation arrayed in fascicles, or with storiform architecture. The spindle cells had oval nuclei, fine chromatin, inconspicuous nucleoli, and bipolar, lightly eosinophilic cytoplasm (Fig. 2A). Admixed with the spindle proliferation was an inflammatory infiltrate containing lymphocytes, plasma cells, and eosinophils. Immunohistochemical analysis showed positive staining for vimentin (Fig. 2B) and desmin, and focal positive staining for smooth muscle actin and cytokeratin (Fig. 2C). The tumor had a low Ki-67 proliferative index. In contrast, the tumor cells were not reactive to CD34, CD99, or S-100 antibodies. The surgical resection margins and all regional lymph nodes were tumor free. Inflammatory myofibroblastic tumor was diagnosed. At the most recent follow-up (12 months after operation), the patient was symptom free and there was no evidence of tumor recurrence on chest CT scan.

## Discussion

IMT is an uncommon pulmonary disease. The incidence rate of IMT among patients with lung resection is 0.04%, and 26% of patients are less than 18 years old [5]. Airway obstruction in IMT, although rare, normally presents at an early stage due to obstructive respiratory symptoms [6]. Most patients are symptomatic. There are respiratory symptoms, such as cough, dyspnea, fever, fatigue, and hemoptysis.

Diagnosis of IMT is difficult to establish before surgery because of its diversified radiologic manifestations and because it can be difficult to distinguish from malignant tumors on small tissue samples obtained from bronchoscopic examination or needle biopsy. In fact, only 6.3% of IMT cases are diagnosed based on analysis of biopsy specimens alone [6]. In addition, IMT is often difficult to differentiate from other neoplasms on PET scan because of the high uptake of tracer in IMT. The prognosis of IMT is dependent on tumor size (less than or equal to 3 cm) and complete surgical resection. The overall 3-year survival rate is about 82% and the overall 5-year survival rate is about 74% [3]. In our case, the tumor was an endobronchial lesion with clear margins. We were unable to prove whether the tumor involved the lung parenchyma.

Surgical management of lesions in the major bronchi is challenging. In our patient, we performed a thoracoscopic technique to cut the adhesion of the major fissure, superior pulmonary vein and pulmonary artery branches to upper lobe of the lung. We then opened the left upper bronchus to confirm that the cut end of the bronchus was free. The bronchus was closed with interrupted sutures.

IMT is characterized histologically by spindle cell proliferation. The tumor is referred to by different names in the literature depending on the predominant cell type encountered in the lesion: plasma cell granuloma or tumor, xanthogranuloma, plasma cell/histiocytoma complex, or post inflammatory pseudotumor [7]. Matsubara et al. used the term inflammatory pseudotumor and described three subgroups based on the cell type most encountered in a mass: organizing pneumonia (44%), fibrous histiocytoma (44%), and lymphoplasmocytic type (12%) [8]. There are regions of organizing pneumonia in all cases, and therefore, the current hypothesis is that IMT might develop in individuals with a past history of upper respiratory infections or pneumonia. Some studies, however, suggest that it might be a true neoplasm as some mutations on chromosome 2p23 of anaplastic lymphoma kinase are found to be related to this tumor [9].

## Conclusions

Although inflammatory myofibroblastic tumor is rare, it should be considered in the differential diagnosis of pulmonary lesions. It is generally a benign lesion, but has potential for local invasion and recurrence. The diagnosis and prognosis are highly dependent on complete surgical resection.

## Consent

Written informed consent was obtained from the patient for publication of this case report and accompanying images. A copy of the written consent is available for review by the Editor-in-Chief of this journal.

## Competing interests

The authors declare that they have no competing interests.

## Authors' contributions

CKC carried out the manuscript. HYF coordinated all authors. CIJ reported pathologic findings and took the pathologic pictures. PRC and HCH collected references; YSL and JST took the pictures of the case report. CYC made conclusion. All authors read and approved the final manuscript.
